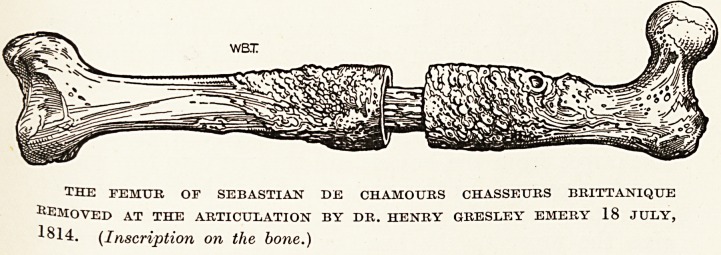# Bristol's Contributions to Medical Progress

**Published:** 1933

**Authors:** 


### BRISTOL'S CONTRIBUTIONS TO MEDICAL PROGRESS.

JOHN FREE is the first of a notable succession of Bristolians who have helped to advance the science and art of medicine. A graduate of Oxford, he came to Bristol, whence, attracted by reports of a revival of learning in Italy, he sailed with three or four friends to ascertain at first hand what new secrets of science the Italian universities could impart. He studied at the Universities of Ferrara, Florence and Padua, settling as a teacher of medicine in the first named. His writings against Diodorus Siculus gained for him the gratitude of the Pope (Pius II), who appointed him to the vacant Bishopric of Bath. To this office he was never consecrated, for he fell ill and died in 1465 before he could return to England. A rapid succession of students followed in Free's footsteps, and Leland's description of him may be recalled with pride: "primus Anglorum erat, qui propulsa barbarie patriam honesto labore bonis literis restituit." He was at some time (1464) Rector of St. Michael the Archangel, Bristol.

Nearly contemporary with John Free was the famous topographer WILLIAM WYRCESTRE (or WORCESTER) (1415–1484), the son of a substantial burgess of Bristol, who after passing four years as a student at Hart Hall in Oxford became a retainer to Sir John Fastolf of Caistre Castle in Norfolk. In process of time he became Fastolf's secretary and physician and finally his executor. Although Worcester is chiefly famed as a chronicler and traveller, he established himself in the decline of his life in Bristol, having a house and garden near St. Philip's Churchyard Gate. There he cultivated medicinal herbs and practised physic till his death about 1484. The MS. of Phreas's (or Free's) *Cosmographia* in the library of Balliol College, Oxford, was presented by Worcester. There appears no other evidence of these two great Bristolians being acquainted.

JOHN MAPLET (1612?–1670), described by Antony à Wood as "learned, candid and ingenious, a good physician, a better Christian and an excellent Latin poet," was at one time Principal of Gloucester Hall in Oxford. He was created Doctor of Physic in 1647. During the Commonwealth he was ejected from his college office, but was restored in 1660. Soon after, however, he resigned. He practised his faculty in the summer-time at Bath and in the winter-time at Bristol. In his *Medicinal Epistles* he refers to the effects of the Bath waters. Some of his observations were embodied in Guidot's treatise on the waters of Bath.

THOMAS DOVER (1660–1742), the pupil and friend of Sydenham, the home-bringer of Alexander Selkirk and the inventor of Dover's Powder, was the first physician appointed in 1696 by the Bristol Corporation of the Poor to take charge of the patients in the newly-opened St. Peter's Hospital. In 1708 he sailed round the world with Woodes Rogers, who brought home Selkirk from Juan Fernandez. It is probable that Dover brought Selkirk to the knowledge of

Daniel Defoe. Dover was the grandson of Robert Dover, who founded the Cotswold Games and is buried at Barton-on-the-Heath. Thomas Dover, who was born at Barton, ended his days at Stanway in Gloucestershire. He died there in 1742, and was buried in the family vault of the Tracys under the altar of Stanway Church. Dover in his life-time was known as the "Quicksilver Doctor." He published a famous book in praise of mercury, *The Physician's Legacy*.

Thomas Lovell Beddoes (1760–1808) was born in Staffordshire in 1760, entered Pembroke College, Oxford, in 1776, studied anatomy in London, and in 1784 commenced a medical career in Edinburgh, whence he graduated in 1787. After this he went to Paris, where he made a friend of Lavoisier. In the autumn of 1787 he assumed the position of Lecturer on Chemistry in Oxford. In 1793 Dr. Beddoes moved to Bristol and practised medicine there. He embarked upon experimental work with gases and introduced the idea of curing diseases such as chlorosis, hypochondriasis, dyspepsia, epilepsy and scurvy by inhalation—as well as (perhaps more successfully) asthma, dropsy and hydrothorax. In 1799 he founded (by public subscription) the Pneumatic Institution at the Hotwells, Clifton, where a hospital of ten beds, a research laboratory, a lecture hall and accommodation for out-patients was provided. He appointed as Director of the Institution Humphry Davy, then aged nineteen years. In the devising of apparatus he was assisted by James Watt.

It was here in April, 1799, that Humphry Davy obtained pure nitrous oxide and demonstrated its effects when inhaled. Humphry Davy was doubtful of the value of the pneumatic treatment of diseases, and did not share Dr. Beddoes's enthusiasm over his apparent cures. In 1801 Humphry Davy left Bristol with the approval of Dr. Beddoes to become Professor of Chemistry at the Royal Institution of London. The value of Davy's discoveries is undisputed. Of Beddoes it has been truly said that he "died disconsolate at his lack of accomplishment." He opposed the slave trade, but Bristol merchants prospered on this traffic for twenty-five years after his death; he had lost his chair at Oxford for championing the cause of the French Revolution; and his Pneumatic Institute failed in its object of curing disease by inhalation, though the discoveries made there by Humphry Davy led presently to nitrous oxide anæsthesia.

Nevertheless, his early efforts at the reduction of infant mortality entitle him to a place in the history of preventive medicine which he failed to win as a therapist, whilst his discovery of Humphry Davy was in the end his greatest achievement of all.

THOMAS BAYNTON (1761–1830) was a Bristol surgeon who saw farther ahead than many men of his own time. His method of strapping ulcers of the leg by overlapping strips of encircling plaster is immortalized in Von Bergmann's *Surgery* as "Baynton's method." A further study of Baynton's writings shows that he was the first surgeon to advocate the treatment of spinal caries by absolute rest in the horizontal position without the aid of setons and issues. His revolt against the accepted barbarities of his day led to most vehement attacks upon him for his temerity by the leading surgeons, particularly Earle and by Estlin in Bristol. Nevertheless, he won the support of wiser and greater men than these, namely Edward Jenner, to whom he dedicated his pamphlet on *The Treatment of Diseases of the Spine*, and Anthony Fothergill, to whom he addressed his *New Method of Treating Old Ulcers of the Legs*.

Baynton was born in 1761 near the foot of Clifton Down. He was apprenticed to Mr. Elmes the apothecary, and became a student at the Bristol Infirmary; he attended the lectures of Pole and Bromfield in London and set up in Bristol to practise in 1782. In 1783 he was an unsuccessful candidate for a surgeon's vacancy at the Bristol Infirmary, and never again presented himself for election. A contemporary (R. Smith) wrote of him: "That he was well acquainted with the great principles of surgery and medicine there can be no doubt, but he was not backward in cherishing the idea that he had outstripped all his brethren in professional acquirements." He made a large income by his practice, and he had other sources of wealth, so that he died worth more than £33,000. Most of this fortune went to the lawyers who contested his long and intricate will after his death in 1830.

James Cowles Prichard (1786–1846), who was Physician to the Royal Infirmary from 1816 to 1843, holds a distinguished place in the annals of psychiatry. His *Treatise on Insanity and Other Disorders Affecting the Mind* was published in 1835, and was for a long time the standard work on the subject. Tuke, in his *History of the Insane in the British Isles* (London, 1882), wrote that Prichard "will always be remembered in the republic of letters by his learned contributions to anthropology and the literature of mental diseases in which he is more especially identified with the doctrine of Moral Insanity." Prichard is acknowledged to have been the first to discriminate that form of mental derangement which is now known as moral insanity or moral imbecility. His eminent position was recognized by his own University of Oxford, who conferred on him the degree of M.D. "by diploma" in 1835, on the occasion of the installation of the Duke of Wellington as Chancellor.

RICHARD BRIGHT (1789 – 1858) was born in Bristol. His father, Richard Bright, was an influential citizen and banker, who lived at Ham Green House. Richard, his third son, was educated at Dr. Estlin's School and subsequently at Edinburgh University and Guy's Hospital. He took the degree of M.D. at Edinburgh in 1813, and in 1820 was appointed to the staff of Guy's Hospital. Ill health caused his retirement from Guy's in 1843 at the early age of 54. The work by which he is chiefly remembered was that on nephritis, in which he correlated the symptoms of renal disease with inflammation of the kidneys. He made many other original observations in clinical medicine, amongst which Garrison in his History of Medicine credits him with the first descriptions of pancreatic diabetes and pancreatic steatorrhœa, acute yellow atrophy of the liver, unilateral convulsions or Jacksonian epilepsy, and (according to Wilks) the cardiac bruit in chorea. Garrison describes him as an accomplished artist, an able botanist and geologist and personally a simple, unprejudiced, truth-loving man.

DR. HENRY GRESLEY EMERY received his medical education at the Bristol Infirmary, and in 1810 applied for the post of Apothecary at the Infirmary, but was not appointed. He served as a Surgeon with the British Army in Portugal, and subsequently practised at Banwell. Concerning the very interesting specimen figured below, which is in the Museum of the Bristol Royal Infirmary, the present Museum catalogue states: "This beautiful specimen is of historic interest. The subject was Sebastian de Chamours, Chasseurs Brittaniques.[1] The limb was removed by Mr. Greiley Emery, July 10th, 1814. This was the first amputation

THE FEMUR OF SEBASTIAN DE CHAMOURS CHASSEURS BRITTANIQUE REMOVED AT THE ARTICULATION BY DR. HENRY GRESLEY EMERY 18 JULY, 1814. (*Inscription on the bone.*)

at the hip-joint performed in England. Mr. Emery had been educated at the Bristol Infirmary." The catalogue was drawn up about 1901 by Mr. E. H. E. Stack, whose custom it was to copy the historical entries without variation from the old manuscript catalogue, now apparently lost. It will be noticed that the entry quoted differs in certain particulars from the inscription on the

[1] "The Chasseurs Britanniques was a Light Infantry Corps, formed early in the Revolutionary War from French Royalists, but in 1811, when it came out to Portugal, recruited entirely from deserters of all sorts . . . Frenchmen, Italians, Poles and Swiss. The officers were almost without exception furious French Royalists, the second generation of the *émigrés*."—Oman, *Wellington's Army* 1809–1814, 1912, p. 225.

bone, the handwriting of which resembles that of Mr. Richard Smith, the founder of the Museum. How, when and where Dr. Emery performed the amputation is not known; nor whether the patient —who, judging by the size of the central sequestrum and the thickness and density of the involucrum, must have been wounded months or years before—survived the operation.

In 1856 DR. WILLIAM BUDD (1811–1880), Physician to the Royal Infirmary from 1847 to 1862, announced his conviction that Typhoid Fever is contagious:—[1]

"This fever has two fundamental characteristics. The first is that it is an essentially contagious disorder; the second, that by far the most virulent part of the specific poison by which the contagion takes effect is contained in the diarrhœal discharges. . . . The discharges from the bowels are thrown into the water-closet or privy. In this way the drains become at once saturated with the specific poison. The poison may give the fever to the healthy in one of three ways: either by percolating into the well which supplies the drinking water; by issuing through defects in the sewer into the air; or by exhaling from the aperture of some ill-trapped closet or privy. . . . This species of fever, like all other contagious fevers, has what is called a period of incubation. I have been led to the conclusion that this period ranges from ten to fourteen days."

Dr. Budd built up a mass of evidence which amounts, as he himself showed, to proof, in a series of articles in the *Lancet*, 1858–59; and in his book on *Typhoid Fever* in 1873.[2] He cites evidence from over a dozen epidemics, many of which have a local interest:

[1] *Lancet*, 6th December, 1856, vol. ii.

[2] *Typhoid Fever*, Longmans, Green & Co., 1873.

for example, in 1842, at the Ashley Hill Orphanage, and in 1847 at Richmond Hill, where all the houses affected drew their water from a contaminated well. His pioneer work on this subject earned the F.R.S., and the rules he laid down in 1858 for disinfection are those in use to-day without any modification.

Yet for many years after he had demonstrated the mode of infection the field was still held by the "pythogenic" theory that typhoid fever arose, as it were, spontaneously from the putrefaction of ordinary sewage. "The doctrine that typhoid fever is a contagious fever and is chiefly propagated by the typhoid excreta is an illusive hypothesis."[1] Murchison as long afterwards as 1867 could be found writing: "There is no proof whatever that the fresh stools passed by a typhoid patient are in any way deleterious. The fever is propagated by the stools only after they have undergone decomposition."[2]

In 1867 Dr. William Budd put forward the then novel theory (which he had first tentatively conceived ten years previously) that Pulmonary Tuberculosis is not a constitutional disorder, but an infectious disease:—[3]

"The following are the principal conclusions to which I have been led regarding phthisis or tubercle:

"1. That tubercle is a true zymotic disease of specific nature, in the same sense as typhoid fever, scarlet fever, typhus, syphilis, etc.

"2. That like these diseases tubercle never originates spontaneously, but is perpetuated solely by the law of contagious succession.

[1] Tanner, *Practice of Medicine*, sixth edition.

[2] Murchison, *British Medical Journal*, 16th March, 1867.

[3] *Lancet*, 1867, vol. ii., p. 451.

"3. That the tuberculous matter itself is (or includes) the specific morbific matter of the disease and constitutes the material by which phthisis is propagated from one person to another and disseminated throughout society.

"4. That the deposits of this matter are therefore of the nature of an eruption and bear the same relation to the disease phthisis as the 'yellow matter' of typhoid fever, for instance, bears to typhoid fever. . . ."

He goes on to support his thesis by citing the heavy incidence in cities, and especially the most crowded and poorest parts, the comparative freedom of rural populations, the often complete freedom of remote peoples, Greenlanders, American Indians and others, until they are brought into contact with Europeans, and the virulent epidemic tuberculosis that ensues when they are, and concludes by advancing proposals for disinfection of sputum as of first importance in the campaign against tuberculosis.

The revolutionary nature of this suggestion at the time when it was made may perhaps not be obvious at the present day. We may recall that in 1883 the first article printed in volume one of this *Journal* was a paper by Dr. Shingleton Smith on "The Proofs of the Existence of a Phthisical Contagion"; and the second article was devoted to clinical evidence *against* that thesis.

AUGUSTIN PRICHARD (1818–1898), the second son of James Cowles Prichard, was Surgeon to the Royal Infirmary from 1850 to 1870, and also for many years to the Eye Dispensary.

In 1850 Mr. Prichard advocated and first performed the operation of enucleation of the eyeball for sympathetic inflammation in the opposite eye :—

"Bristol Royal Infirmary. Cases under Augustin Prichard, Esq.[1]

"James F., 46, admitted September, 1850. Thirteen years previously he had met with an accident . . . a piece of iron entered his left eye . . . and the constantly recurring inflammation of the injured eye has kept up so much sympathetic inflammation in the right that he has been for a long time disabled from gaining his livelihood. . . . He is quite unable to use the right eye from the great intolerance to light. . . . (After several months' treatment) the symptoms still remained in all their severity and . . . I proposed as a last resource the removal of the globe of the eye. . . . I performed the operation in the usual way on the 14th of October, the patient being under the influence of chloroform. . . .

"October 23rd, went out quite well, his right eye being quite strong and the sight good. The case is interesting as being the first recorded instance as far as I can discover in which extirpation of the globe has been performed for a disease of the eye which is not malignant. I should think that a similar operation might be performed in many instances with advantage . . . when the sound eye is endangered, as it undoubtedly is, by the disease of the other."

Sir J. H. Parsons, writing on the subject of "Sympathetic Ophthalmia,"[2] says :—

"The most notable contribution to our knowledge of this disease was made by Augustin Prichard (1851) of Bristol, who first proved the efficiency of excision of the exciting eye. This treatment was received with distrust, even by von Graefe. . . . von Graefe later recognized the value of excision."

[1] *The Provincial Medical Journal,* 1851, p. 66.

[2] *Pathology of the Eye,* vol. iv., p. 1,229.

DR. WILLIAM BIRD HERAPATH, F.R.S. (1820–1868), was the son of William Herapath (1796–1868), who was one of the founders of the Bristol Medical School, Professor of Chemistry and Toxicology, 1828–1867, and a renowned toxicologist.[1] The son also distinguished himself in the same speciality, and since they both died in the same year some confusion between them has arisen. Dr. W. B. Herapath entered the Medical School in 1839, became M.R.C.S. 1844, M.D. 1851, and practised in Old Market Street. He discovered the sulphate of iodo-quinine[2] ("Herapathite") and developed from this discovery the test for the presence of quinine in small quantities.[3] "Having been struck with the facility of application and the extreme delicacy of the reaction of polarised light . . . upon the sulphate of iodo-quinine, I determined upon attempting to bring this method into practical use for the detection of minute quantities of quinine in organic fluids. I have at length discovered a process by which it is possible to demonstrate the presence of quinine even if in quantities not exceeding 1/100,000 part of a grain. The same process (modified) has enabled me to prove that quinidine (is excreted) by the kidnies unaltered . . . which has not hitherto been observed."

ELIZABETH BLACKWELL (1821–1910), the pioneer of women doctors, was born in Bristol on 3rd February, 1821, the daughter of Samuel Blackwell, who

[1] Cf. *Scholæ Med. Bristol*, p. 6; John Wright & Sons Ltd., 1933.

[2] "On the optical properties of a newly-discovered salt of quinine," *Philosophical Magazine*, March, 1852, p. 161; Sept., 1852, p. 186.

[3] "On the discovery of quinine . . . in the urine of patients . . ." *Ibid.*, 1853, p. 171.

emigrated with his family to America in 1832. She was educated at private schools in Bristol and New York, and herself taught in Kentucky and in North and South Carolina. Presently she became a student of medicine in the University of Geneva, U.S.A. She also studied in Paris and, thanks to the help of Sir James Paget, at St. Bartholomew's in London. She practised in New York from 1851, founding there a hospital and medical school for women. She also founded the National Health Society of London and assisted in founding the London School of Medicine for Women. Dr. Blackwell was the first woman admitted to the Medical Register of the United Kingdom. She died at Hastings 31st May, 1910.

ROBERT FLETCHER (1823–1912) was born in Bristol and entered the Medical School in 1839. In 1844 he qualified as M.R.C.S. and L.S.A. In 1847 he went to the United States, settling in Cincinnati, where he practised medicine for some years. He served as an army surgeon in the Civil War and was given the rank of colonel for meritorious service. From that time forward he worked at Washington in the Surgeon-General's Department. In 1879, when the *Index Medicus* was first published, Dr. Fletcher was associated with Dr. Billings as co-editor for twenty-one years and as Editor-in-Chief for nine years. In 1906 he was given a banquet by the leading members of the profession in the United States in recognition of his work at the Surgeon-General's Library. In 1910 the Royal College of Surgeons of England awarded him their gold medal, and in 1912 he received the honorary degree of M.D. from the University of

Bristol. The Editorial Note in this Journal referred to his death as depriving the Bristol Medical School and Royal Infirmary of their oldest student and the world of medicine of its Bibliographer-in-Chief.

Of J. Greig Smith (1854–1897) the *Journal of the American Medical Association*[1] said: "His treatise on *Abdominal Surgery* published in 1887 placed him in the front rank." And it is probably as the author of this work that he is known to the world at large, though to us his best claim is that he was the first Editor of the *Journal* and the founder of the Society's Library. The story is told at greater length in the History of the *Journal* in this issue. Greig Smith himself ranked as his best work his contributions to the pathology of bone, particularly "The Pathology and Treatment of Chronic Osteo-arthritis"[2] and "On the Formation of Callus,"[3] and it was in recognition of these original researches that the Edinburgh Royal Society elected him to their Fellowship. The impulse he gave to the progress of surgery in his day came from his own first-hand knowledge, acquired in the course of his many and varied researches in the problems of surgery and pathology—researches the publication of which was prevented by his premature death at the age of 43.

In 1892 Mr. A. F. Stanley Kent, Professor of Physiology in University College and the University of Bristol 1899–1918, demonstrated the auriculo-

[1] August 7th, 1897, p. 297.

[2] Bristol Royal Infirmary Reports, vol. i., 1878–79, p. 81.

[3] *Journal of Anat. and Physiology*, vol. xvi., 1881–82, p. 153.

ventricular bundle of the mammalian heart, sometimes called the bundle of His. Up to that time it had been thought that the fibrous tissue of the mammalian heart completely separated the muscular tissue of the auricles from that of the ventricles, and several ingenious hypotheses were advanced to explain the passage of the systolic contraction wave from one to the other.[1] "A series of rats was taken and sections were prepared as a complete series right through the heart. The result of a careful study of these series has been to convince me that even in the adult animal a strongly-marked band of muscular tissue remains and preserves the integrity of the muscular connection between the two chambers of the heart. . . . But in addition to this comparatively simple mode of connection, we have, in the monkey, remarkably well developed, a second and far more complicated system of communicating fibres. Lying on the borderland between the undoubted muscle on the one hand and the connective tissue on the other, there may be distinguished cells which whilst certainly not belonging to the connective tissue yet differ very markedly from the muscle fibres in the immediate neighbourhood. These cells are usually spindle-shaped, nucleated, granular and often transversely striated, and are obviously a form of tissue intermediate between ordinary cardiac tissue and plain or non-striped muscle."

At the foundation of many of the Bristol hospitals medical men have played important parts :—

[1] *Journal of Physiology*, vol. xiv., 4, 5, 1893, p. 217.

DR. BONYTHON was a most active promoter of the Bristol Infirmary, and might almost claim to be a co-founder with John Elbridge. His is the first name on the roll of Infirmary physicians, where he held office from 1737 to 1761.

MR. W. H. GOLDWYER, "Surgeon and Oculist," with great energy enlisted the sympathies of many eminent citizens in a scheme which he launched in 1810 for founding an Eye Hospital. This proved an immediate success, and Goldwyer's services were so generally appreciated that the freedom of the city was presented to him in 1816. The success of the Bristol Eye Hospital has proved to be enduring, and at the present time it is undergoing important extension.

JOHN BISHOP ESTLIN (whose sister married James Cowles Prichard) founded in 1812 the Bristol Eye Dispensary, and it was at this Institution that Augustin Prichard obtained his first experience of ophthalmic surgery, which he afterwards used to such good purpose.

DR. KENTISH was the most active medical man amongst the promoters of the General Hospital. He had been Physician to St. Peter's Hospital for twenty-eight years and to the Bristol Dispensary for the same period. He had also been the proprietor of the "Vapour Baths" in College Green, opposite the east end of the Cathedral. The Bristol General Hospital was formally opened in 1832. Kentish was for a long period a member of the Infirmary

Committee, and no one realized more fully how inadequate the one institution was to the city's needs.

There was also a DR. ABRAHAM BAGNELL, who was greatly interested in the foundation of the General Hospital. His pamphlet urging its claims has been preserved in part by Munro Smith in his *History of the Bristol Royal Infirmary.* Bagnell attacked the inhuman practices of the Infirmary Staff, speaking with horror of "an instrument called the stethoscope," which "is applied to different parts of the chest and sides of the palpitating patient." He described his pamphlet as an appeal to humanity and benevolence.

The year 1833 was remarkable in Bristol not only for the foundation of the Medical School, but because the first Annual Meeting of the Provincial Medical and Surgical Association was held here on 19th July of that year. The Editor of the *Lancet,* in reviewing that meeting, criticized the name, and suggested "British Medical Association," which was afterwards adopted.

"PROVINCIAL MEDICAL AND SURGICAL ASSOCIATION."[1]

"Yesterday our Infirmary was complimented with the distinguished honour of being the place selected for holding the first anniversary meeting of the above Society, in consequence of the very numerous list of Bristol members, and the respectable support given to the Association by the most eminent professional gentlemen of this city."

[1] *Lancet,* 1832–33, vol. ii., p. 572, 27th July, 1833.

Of the 400 members 50 were Bristolians; 200 attended the meeting. Dr. Carrick was unanimously called to the Chair (his speech is recorded in full) and no less than eight Bristol medical men were elected to the Council of the Association.

## Figures and Tables

**Figure f1:**